# Roles of computed tomography and [^18^F]fluorodeoxyglucose-positron emission tomography/computed tomography in the characterization of multiple solitary solid lung nodules

**DOI:** 10.3332/ecancer.2012.266

**Published:** 2012-08-28

**Authors:** LL Travaini, G Trifirò, PD Vigna, G Veronesi, TM De Pas, L Spaggiari, G Paganelli, M Bellomi

**Affiliations:** 1 Division of Nuclear Medicine, Development and Clinical Pharmacology Unit, European Institute of Oncology, Milan, Italy; 2 Division of Radiology, Development and Clinical Pharmacology Unit, European Institute of Oncology, Milan, Italy; 3 Division of Thoracic Surgery, Development and Clinical Pharmacology Unit, European Institute of Oncology, Milan, Italy; 4 Division of New Drugs, Development and Clinical Pharmacology Unit, European Institute of Oncology, Milan, Italy; 5 School of Medicine, University of Milan, Milan, Italy; 6 Nuclear Medicine Unit, Fondazione Salvatore Maugeri, Pavia, Italy

## Abstract

The purpose of this study is to compare the performance of multidetector computed tomography (CT) and positron emission tomography/CT (PET/CT) with [^18^F]fluorodeoxyglucose in the diagnosis of multiple solitary lung nodules in 14 consecutive patients with suspicious lung cancer. CT and PET/CT findings were reviewed by a radiologist and nuclear medicine physician, respectively, blinded to the pathological diagnoses of lung cancer, considering nodule size, shape, and location (CT) and maximum standardized uptake value normalized to body weight (SUVbw max). Nodules were judged malignant or benign. The sensitivity, specificity, and accuracy of the two techniques were compared. CT had a sensitivity, specificity, and accuracy of 93.7, 86.7, and 90.3%, respectively, whereas PET/CT had a sensitivity, specificity, and accuracy of 75, 100, and 87.1%, respectively. Clinical management would have been erroneous in two patients by CT alone and in four patients by PET/CT alone. In one patient, the two techniques misdiagnosed the nodules (2 CT and 1 PET/CT). CT and PET/CT have complimentary roles in characterization of multiple solitary pulmonary nodules. Small nodules are poorly characterized by CT, and small-sized low-SUV malignant nodules are difficult to detect with PET/CT.

## Background

Solitary pulmonary nodules (SPNs) are spheroid, well-circumscribed, and radiographic opacities up to 3 cm in diameter completely surrounded by aerated lung, with atelectasis, hilar enlargement, and pleural effusion absent [1, 2]. SPNs are a common incidental finding on thoracic X-ray and computed tomography (CT) and are malignant in 10–20% of cases. The presence of risk factors (mainly smoking) markedly increases the probability of malignancy, while provenance from certain geographic regions increases the risk that a nodule is benign (e.g. tuberculosis related). In addition to size, other nodule features, such as size increase or density change on repeated scan, relation to adjacent thoracic structures, and peripheral vs. central position, also influence the probability of malignancy.

In recent years, several studies have assessed the feasibility of screening high-risk populations with low-dose CT to identify SPNs [3–5] which may be further investigated by [^18^F]fluorodeoxyglucose (FDG) positron emission tomography (PET) or combined PET/CT. Malignant SPNs identified by screening are very often at stage I and have excellent five-year survival after surgery. CT and PET/CT are also used to investigate Solitary pulmonary nodules in persons with a previous history of cancer, which are best considered malignant until shown to be benign [4].

Computed tomography provides information on nodule size, shape, density, homogeneity, contrast enhancement, and relations with adjacent mediastinal and pleural structures. CT may also reveal enlarged mediastinal lymph nodes with a high probability of metastatic involvement [6].

[^18^F]fluorodeoxyglucose- positron emission tomography reveals increased glucose metabolism, which is a major criterion for distinguishing malignant from benign lesions, and has higher accuracy than CT in detecting lymph nodal involvement [7].

Most of the literature has concentrated on solitary lung nodules [8, 9]. However, it is not uncommon to detect several solitary lung nodules in a single patient. The malignant vs. benign nature of all nodules must be ascertained prior to surgery if possible, particularly if located in different lung lobes. This represents a considerable diagnostic challenge. In the present study, we compared the performance of multidetector CT with FDG-PET/CT in the characterization of multiple solitary lung nodules in a consecutive and heterogeneous series of patients, some with a previous history of cancer.

## Methods

We performed a search in the database of the Thoracic Surgery Division through the association of PET/CT and CT carried out in our institution in a series of 2,117 consecutive patients with solid lung nodules. From July 2003 to December 2006, the data of 14 patients with two or more nodules >7 mm were obtained and were re-examined by an experienced radiologist and an experienced nuclear medicine physician, both blinded to the histological status of the nodules.

Six of the 14 patients had a history of cancer: 1 lung, 3 colon, 1 kidney, and 1 breast; and the nodules were identified during follow-up. In the remaining 8 patients, 7 of whom were smokers or former smokers, and the nodules were diagnosed incidentally during investigation for other non-oncological conditions. The patients were classified as at high-, intermediate-, or low-risk for cancer. High-risk patients had a previous history of cancer; low-risk patients were non-smokers, age below 45 years and with rounded nodules of diameter less than 15 mm; all others were considered intermediate risk [10].

### Computed tomography

Scans were obtained with a 16-slice GE Light Speed CT (GE Medical Systems, Milwaukee, WI) with the following acquisition parameters: collimation 20 mm, reconstructed slice thickness 2.5 mm; standard reconstruction filter; 120 kVp, 220–400 mA (automatic exposure); rotation time 0.8 s; speed 18.75 mm/rot (pitch 0.938). Scans began 50 s after i.v. injection of 1.5 ml/kg of contrast containing 350–370 mg I/ml, at 2 ml/s, followed by 50 ml of saline at 2 ml/s.

The radiologist examined the CT scans, visually assessing lesion size, smooth vs. irregular margins, round vs. oval shape, lesion homogeneity and expressed the result as benign or malignant [11]. The results were classified as true positive (TP), true negative (TN), false positive (FP), or false negative (FN) in relation to the histologic findings. Sensitivity (TP/TP+FN), specificity (TN/TN+FP), and accuracy (TP+TN/whole population) were then calculated.

### Positron emission tomography/computed tomography

In order to improve image quality and to suppress myocardial glucose utilization, patients were asked to refrain from eating carbohydrates the day before the examination. On the day of the examination, serum glucose was measured and found to be below 150 mg/dl in all cases. Five MBq/kg of [^18^F]FDG (supplied and quality controlled by an external pharmacy) was administered i.v., under fasting conditions. Images were obtained using a PET/CT scanner (Discovery LS; GE Medical Systems, Waukesha, WI). CT settings were 120 kVp, 80 mA, 5 mm scan width, 3.87–4.25 mm interval in high-sensitivity mode with table speed 15 mm per rotation. Whole body scans were acquired from the pelvis to the head in the supine position for a scan time of 24–28 min (4 min per bed position) in 2D mode. Emission data were reconstructed using iterative algorithms and corrected for attenuation using transmission data derived from the CT. Attenuation corrected images were reconstructed in axial, coronal, and sagittal planes.

The nuclear medicine physician was informed of the presence and position of lung nodules, but was blinded to their pathological status. The images were interpreted visually and semi-quantitatively by the measurement of maximum standardized uptake value, normalized to body weight (SUVbw max), on axial images, using the corresponding CT image with lung window (reference image) to confirm location. Semi-quantitative analysis using SUV was also performed in order to reduce the influence of subjective operator factors.

Because of the mainly vertical respiratory excursion, SUVbw max was also measured for four slices up and four slices down from the reference image. Nodules were considered malignant for SUVbw max ≥2.0, benign for SUVbw max <2.0, and classified in relation to the pathological findings.

Some patients were included in a clinical protocol approved by our ethical committee. These subjects gave informed consent to the study.

## Results

### Pathology

The characteristics of the lung nodules found in each patient are shown in Table 1. Mean pathological size was 13.2 mm (SD 12.09 mm; range 4–69 mm). Mean pathological size of the benign nodules (*n* = 15) was 8.07 mm (SD 3.95; range 4–17 mm). Mean pathological size of the malignant nodules (*n* = 16) was 18.73 mm (SD 15.1; range 5–69 mm). Of the benign nodules, 14 were hamartomas and 1 was a focus of chronic inflammation. Of the malignant nodules, 10 were primary lung cancer (mean size 22.4 mm; SD 17.6; range 10–69 mm) and 6 were metastasis (mean size 11.4 mm; SD 6.5; range 5–20 mm). The performances of CT and PET/CT are compared in Table 2.

### Computed tomography

Computed tomography was performed a mean 16 days (range 0–46) before surgery. Thirty-one nodules were identified in the 14 patients: 21 in the right lung (10 upper lobe, 2 middle lobe, and 9 lower lobe) and 10 in the left lung (8 upper lobe and 2 lower lobe). Margins were smooth in 16, irregular in 15. Margins were irregular in 14/17 (82.3%) malignant nodules and smooth in 13/15 (86.7%) benign nodules. 13/31 (41.9%) nodules were homogeneous: 9/15 (60%) benign nodules were homogeneous and 12/16 (75%) malignant nodules were non-homogeneous. Shape was round in 11 and oval in 19: 6/16 malignant lung nodules were round and 10/16 were oval; 5/15 benign nodules were round and 10/15 oval.

**Figure 1:**
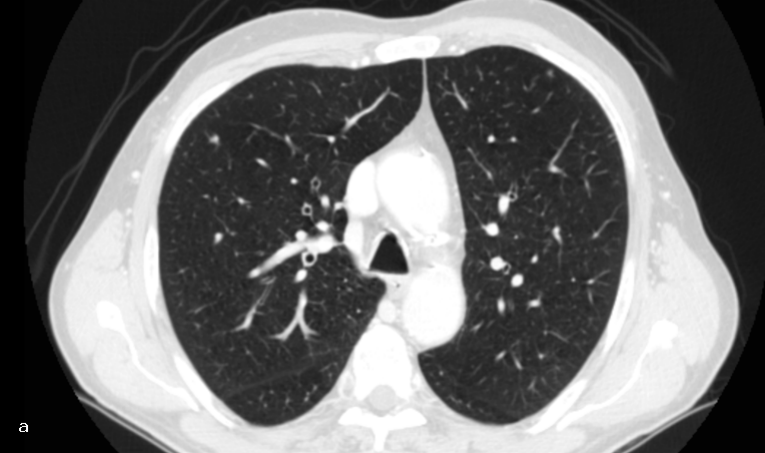
Lung chronic inflammation. Patient #8. Lung chronic inflammation in the right upper lobe. (a) CT recognized the lung nodule as malignant (FP CT) and (b) PET/CT did not reveal increased FDG in relation to the size of the nodule (TP PET/CT).

**Figure 2:**
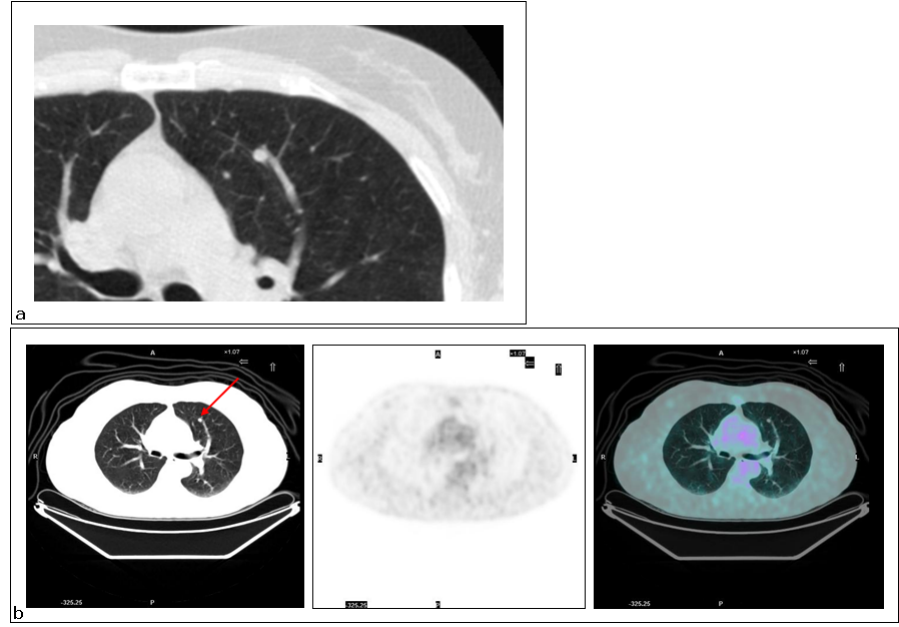
Lung metastasis. Patient #11. Lung metastasis in the upper left lobe. (a) CT interpreted it as benign (FN CT) as did (b) PET (FN PET/CT).

Seventeen (54.8%) nodules were judged malignant and 14 (45.2%) benign. Two nodules considered malignant were found to be benign (2 FP) (Figure 1) and one nodule considered benign was malignant (1 FN) (Figure 2).

### Positron emission tomography/computed tomography

Positron emission tomography/computed tomography was performed a mean of 15 days (range 0–45) before surgery. Twelve nodules had SUVbw max above cut-off (mean 8; range 2.7–16); all were malignant at definitive pathology. The remaining lung nodules (*n* = 19) had SUVbw max <2.

**Figure 3:**
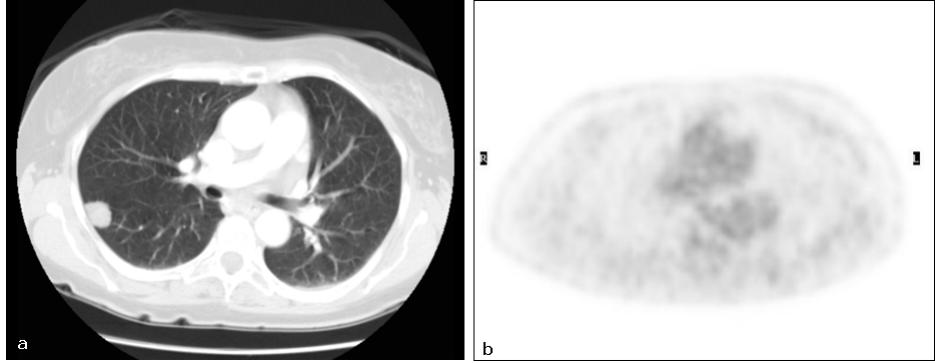
Primary lung cancer. Patient #14. Lung cancer in the right lower lobe as detected by (a) CT (TP CT) and (b) PET/CT did not reveal increased FDG uptake (FN PET/CT).

**Figure 4:**
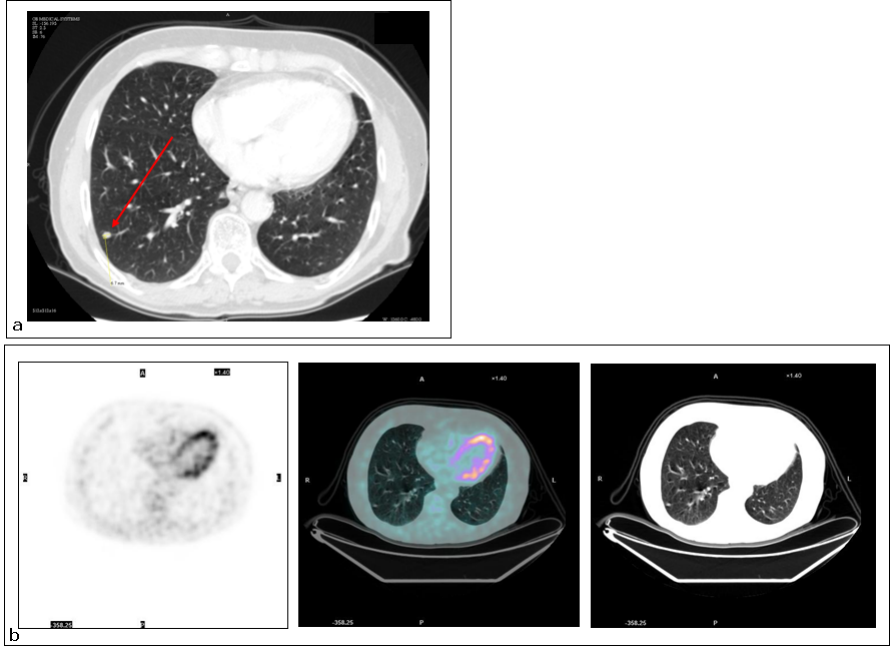
Lung metastasis. Patient #13. Lung metastasis in the right lower lobe. (a) CT detected the malignant nodule (TP CT), while (b) PET did not (FN PET/CT).

There were four FN findings on PET: two had low-glucose metabolism (Figure 3), one a metastatic lesion from kidney cancer of 5 mm in maximum diameter, and the other a primary lung cancer with prominent mucinous component, 20 mm in diameter (Figure 4).

### Diagnostic accuracy

Nine patients were correctly diagnosed by both techniques, all of whom had only two solitary lung nodules. For one case only (patient 11 with two nodules), both nodules were wrongly diagnosed: the malignant nodule was considered benign by both techniques, and the benign nodule was considered malignant by CT and benign by PET/CT. With regard to the three patients with three solitary nodules, in each case one nodule was diagnosed erroneously by one of the two techniques (1 FP by CT; 2 FNs by PET/CT); in each case, the malignancy was also present in a lobe containing other nodules and was removed. In one of these patients (patient 8), the third nodule was FP by CT and TN by PET/CT, which on resection was found to be a focus of chronic inflammation in a lobe harbouring NSCLC. In patients 9 and 13 with three nodules, the third nodule was FN by PET/CT and TP by CT; in each case, the nodule was a metastasis from lung cancer in another lobe. In both these cases, the second nodule was correctly identified as benign by both the techniques.

Seven patients had two solitary lung nodules in the same lobe; in three of these (patients 3, 6, and 7), both nodules were correctly diagnosed by both techniques; in the other four cases (patients 8, 9, 13, and 14), one nodule was misdiagnosed, with three FNs by PET/CT while CT was TP, and one FP by CT while PET/CT was TN.

Seven patients were classified as high risk and seven as intermediate risk. In the high-risk group, sensitivity and specificity were 87.5 and 83.3%, respectively, for CT, and 62.5 and 100%, respectively, for PET/CT. In the intermediate-risk group, sensitivity and specificity were 100 and 88.9%, respectively, for CT, and 87.5 and 100%, respectively for PET/CT.

## Discussions

Data from the literature mostly concern isolated SPNs and their impact on patient management [8, 9]. In the management of pulmonary nodules, surgeons strive to maximize resection rates for early malignant lesions, while avoiding surgery for benign pulmonary nodules, it become crucial to recognize the malignant nature of the nodule and to distinguish it from the benign one.

In the present study, we assessed the performance of multidetector CT in comparison to PET/CT in a heterogeneous group of patients in the evaluation of two or more SPNs. We found that CT and PET/CT had comparable diagnostic accuracy overall (90.3 vs. 87.1%) in the intermediate-risk group (both 94.1%), whereas PET/CT performed worse in the high-risk group (85.7 vs. 78.6%). PET/CT diagnosed all benign lesions correctly (specificity 100%), whereas CT misdiagnosed a 6-mm hamartoma and a 12-mm focus of inflammation as malignant (specificity 87.5%).

Positron emission tomography/computed tomography was not as effective as CT at diagnosing malignant nodules in this series, as also reported in a previous study [12]. It is worthwhile examining the reasons for the FNs on PET/CT [13, 14]. They were a 5-mm kidney cancer metastasis (patient 11), also considered benign by CT; a 20-mm primary lung adenocarcinoma with mucinous and bronchoalveolar component (patient 14) and two metastases from colorectal cancer of 7.5 mm (patient 9) and 5 mm (patient 13). Thus, two of the four FNs were of a histological type known for low rate of glucose metabolism. The 5-mm diameter metastasis was in the right inferior lobe where movement can easily lead to underestimation of glucose metabolism. The misdiagnosis of the 7.5-mm lesion can only be interpreted as operator error, since SUV was >2. The ‘mitigating’ aspect for patients 9 and 13 is that both had another metastatic lesion, correctly identified by CT and PET/CT. These findings add further weight to the opinion that a semi-quantitative approach does not improve PET/CT accuracy over visual analysis for solid pulmonary lesions characterized by low-FDG uptake, which nonetheless have a low probability of being malignant [15].

Although multiple and isolated SPNs have similar causes, for multiple nodules, metastasis is the most likely malignant diagnosis and active infectious or inflammatory granulomatous disease the most likely benign cause [16]. The frequency of multiple SPNs varies with patient characteristics. In a Japanese study [17], 10% of patients with suspected lung cancer had a second nodule during work-up, and 60% of these were benign at surgery. Keogan *et al* [18] reported a second small non-calcified pulmonary nodule on CT in 16% of patients with operable clinical stage I to IIIA NSCLC. The nodules ranged from 4 to 12 mm in diameter: 70% were benign and 11% malignant on pathological examination, with status undetermined in 19%.

Multiple SPNs are a frequent finding in screening studies. In the Early Lung Cancer Action Project [19], 30% of cancer patients had additional lung nodules characterized as benign on follow-up. In the Mayo Clinic Screening Study [20], over 50% of the 31 volunteers with prevalent cancers had other nodules, all but one of which proved to be benign by absence of growth during follow-up.

In patients with extra-pulmonary cancer, an SPN can be a metastasis, primary lung cancer, or benign disease [21]. Metastasis should not necessarily preclude surgery, since 5-year survival after metastasectomy can be up to 80% for patients with germ cell tumours, 53% for gynaecologic cancers, 44% for head and neck cancer, 43% for renal cell carcinoma, 38% for colon cancer, 34% for sarcoma, 34% for breast cancer, and 16% for melanoma [22]. As discussed by Benjamin *et al* [23], the reported malignancy rate for SPNs in patients with extrathoracic malignancy varies widely, from 13 to 87% for small pulmonary nodules. In the study of Ginsberg *et al* [24] in which small nodules (1 cm) detected on CT were removed by video-assisted thoracoscopic surgery, the malignancy rate was 42% in patients with prior malignancy. Nevertheless the literature data indicate, as expected, that small nodules in patients with known primary malignancies have a higher malignancy rate than those in patients without known primary malignancies.

[^18^F]fluorodeoxyglucose-positron emission tomography and [^18^F]fluorodeoxyglucose-positron emission tomography/computed tomography PET/CT have been widely used to characterize indeterminate lung nodules identified on CT, with sensitivities ranging from 88 to 96% and specificities ranging from 78 to 92% [25]. Yi *et al* [13] reported that FDG-PET/CT was more sensitive and accurate than dynamic helical CT for the detection of malignancy and proposed PET as the first-line method for evaluating SPN. However, in the oncologic population, Reinhardt *et al* [14] report a PET sensitivity of 40 and 78% in pulmonary metastases ranging from 5 to 7 mm and from 8 to 10 mm, respectively. The article by Grgic *et al* [26] found that lesion SUV together with pre-test probability assessment could stratify the risk of individual risk of malignancy. The authors also found that higher FDG uptake in lung cancer nodules correlated with poorer survival.

Unfortunately, no data are available regarding the FDG uptake of metastatic lung nodules. However, in this context, FDG-PET can identify extra-pulmonary disease, which usually contraindicates thoracic surgery for metastatic lung nodules.

Comparison of the performance of CT and PET/CT relative to pathological findings in a small heterogeneous series of patients with multiple SPNs

## Conclusions

Our study, although heterogeneous and characterized by a small number of pts, suggests that CT and PET/CT may play complementary roles in the characterization of multiple SPNs. It also indicates that their management needs to be carefully evaluated in a multidisciplinary setting taking into account clinical data, risk factors, and the results of both diagnostic techniques.

## Figures and Tables

**Table 1:** CT and PET/CT findings in relation to histological findings.

	First nodule					Second nodule				Third nodule			
Pt	Histology	CT	PET/CT	Site	Histology	CT	PET/CT	Site	Histology	CT	PET/CT	Site
1	NSCLC (spinocellular)	TP	TP	RUL	Hamartoma	TN	TN	RLL				
2	NSCLC (adenocarcinoma)	TP	TP	LML	Hamartoma	TN	TN	RUL				
3	NSCLC (spinocellular)	TP	TP	LUL	Hamartoma	TN	TN	LUL				
4	Metastasis	TP	TP	RLL	Hamartoma	TN	TN	LUL				
5	NSCLC (spinocellular)	TP	TP	RUL	Hamartoma	TN	TN	LUL				
6	NSCLC (adenocarcinoma)	TP	TP	RUL	Hamartoma	TN	TN	RUL				
7	NSCLC (adenocarcinoma)	TP	TP	LUL	Hamartoma	TN	TN	LUL				
8	NSCLC (adenocarcinoma)	TP	TP	RUL	Hamartoma	TN	TN	RLL	Chronic Inflamm.	**FP**	TN	RUL
9	Metastasis	TP	TP	RUL	Hamartoma	TN	TN	RLL	Metastasis	TP	**FN**	RUL
10	NSCLC (adenocarcinoma)	TP	TP	LUL	Hamartoma	TN	TN	LIL				
11	Metastasis	**FN**	**FN**	LUL	Hamartoma	**FP**	TN	LIL				
12	NSCLC (adenocarcinoma)	TP	TP	RLL	Hamartoma	TN	TN	RUL				
13	Metastasis	TP	TP	LML	Hamartoma	TN	TN	RLL	Metastasis	TP	**FN**	RLL
14	NSCLC (BAC/mucinous)	TP	**FN**	RLL	Hamartoma	TN	TN	RLL				

*Pt* patient, *NSCLC* non-small cell lung cancer, *BAC* bronchoalveolar carcinoma, *TP* true positive, *TN* true negative, *FN* false negative, *FP* false positive, *RLL* right lower lobe, *RUL* right upper lobe, *LIL* left lower lobe, *LML* left middle lobe, *LUL* left upper lobe

**Table 2:** Comparison of CT and PET/CT performance.

	TP	TN	FP	FN	Sensitivity (%)	Specificity (%)	Accuracy (%)
PET/CT	12	15	0	4	75.0	100.0	87.1
CT	15	13	2	1	93.7	86.7	90.3

*TP* true positive, *TN* true negative, *FN* false negative, *FP* false positive
